# Clomiphene-Induced Portal Vein Thrombosis and Superior Mesenteric Venous Thrombosis

**DOI:** 10.7759/cureus.70627

**Published:** 2024-10-01

**Authors:** Ayesha Fatima, Liam Joseph Fernyhough, Abdul Moqeeth Mohammed, Vajeeha Haider, Asma Eltahir

**Affiliations:** 1 Family Medicine, Primary Health Care Corporation, Doha, QAT; 2 Hematology and Medical Oncology, Weil Cornell Medical College, Doha, QAT; 3 Family Medicine, Hamad Medical Corporation, Doha, QAT; 4 Internal Medicine, Hamad Medical Corporation, Doha, QAT; 5 Pharmacy, Hamad Medical Corporation, Doha, QAT

**Keywords:** clomiphene-induced thrombosis, male infertility treatment, portal vein thrombosis (pvt), risk factors for thrombosis, superior mesenteric vein thrombosis

## Abstract

Clomiphene citrate is a selective estrogen receptor modulator commonly used off-label for male infertility despite being approved only for female infertility. Clomiphene is generally safe, and serious adverse effects such as venous thrombosis are rarely reported. This report presents the case of a 32-year-old man who developed portal vein thrombosis (PVT) and superior mesenteric venous thrombosis (SMVT) following three months of clomiphene therapy for infertility. The patient presented with severe abdominal pain and was found to have acute thrombosis on CT imaging. No other underlying hypercoagulable condition was present, and clomiphene was identified as a possible provoking factor for the thrombosis. The patient was successfully treated with anticoagulation therapy and was discharged on dabigatran for six months, with follow-up imaging planned. This is the first reported case linking clomiphene use to PVT and SMVT. Further studies are necessary to better understand the degree of risk and mechanisms underlying clomiphene-induced thrombosis.

## Introduction

Clomiphene citrate (CC) is a selective estrogen receptor modulator approved by the U.S. Food and Drug Administration (FDA) for the treatment of female infertility [1]. The FDA has not authorized it for male infertility, but off-label use in male patients has increased [2,3]. It is now commonly used to treat men with infertility and is considered safe, with only rare occurrences of serious side effects [4]. More common clomiphene risks and adverse effects include hot flashes, mood swings, blurred vision, nausea, vomiting, and headaches [4]. Venous thromboembolism (VTE) is a serious adverse effect rarely associated with clomiphene use [2,5].

There is no previously reported case of portal vein thrombosis (PVT) or superior mesenteric venous thrombosis (SMVT) as a clomiphene-related adverse event in a young adult. This rare manifestation of venous thrombosis has prompted us to report the data to the scientific community.

## Case presentation

A 32-year-old male patient presented to the emergency department with generalized abdominal pain for one month. The pain was initially mild but worsened over three days prior to presentation when the pain became severe, at 9/10 on the pain scale, to the point where he could not sleep. The pain was aggravated by lying flat and eating meals. Avoiding meals relieved the pain. He had not passed stools for three days. There were no other associated symptoms of fever, nausea, vomiting, diarrhea, chest pain, or back pain. There was no recent travel. The medication history was significant for three months of treatment with CC for infertility. He did not have any other past medical or surgical history. The initial laboratory investigations are given in Table 1. On abdominal examination, there was diffuse abdominal tenderness, particularly in the right lower quadrant.

**Table 1:** Initial laboratory investigations WBC, white blood cells; PT, prothrombin time; ALT, alanine aminotransferase; AST, aspartate aminotransferase; ALP, alkaline phosphatase; CRP, C-reactive protein

Parameters	Values	Reference Range
WBC	8.4 x 10^3^/uL	4.0-10.0 x 10^3^/uL
Hemoglobin	15.3 g/dL	13-18 g/dL
Platelets	224 x 10^3^/uL	150-400 x 10^3^/uL
PT	14.7 secs	9.4-12.5 secs
D-dimers	2.05 mg/L	0.0-0.49 mg/L
ALT	31 U/L	0-41 U/L
AST	25 U/L	0-41 U/L
ALP	63 U/L	40-129 U/L
CRP	16.4 mg/L	0.0-5.0 mg/L
Lactic acid	1.3 mmol/L	0.5-2.2 mmol/L
Amylase	28 U/L	13-53 U/L
Creatinine	119 umol/L	62-106 umol/L
Urea	3.7 mmol/L	2.5-7.8 mmol/L
Total protein	66 gm/L	60-80 gm/L
JAK-2 PCR	Negative	-
Prothrombin gene mutation PCR	Negative	-
Factor V Leiden	Negative	-
Lupus Anticoagulant	Negative	-
Anticardiolipin antibodies	Negative	-
Anti-B2 glycoprotein antibodies	Negative	-
Antinuclear antibodies	Negative	-

**Figure 1:**
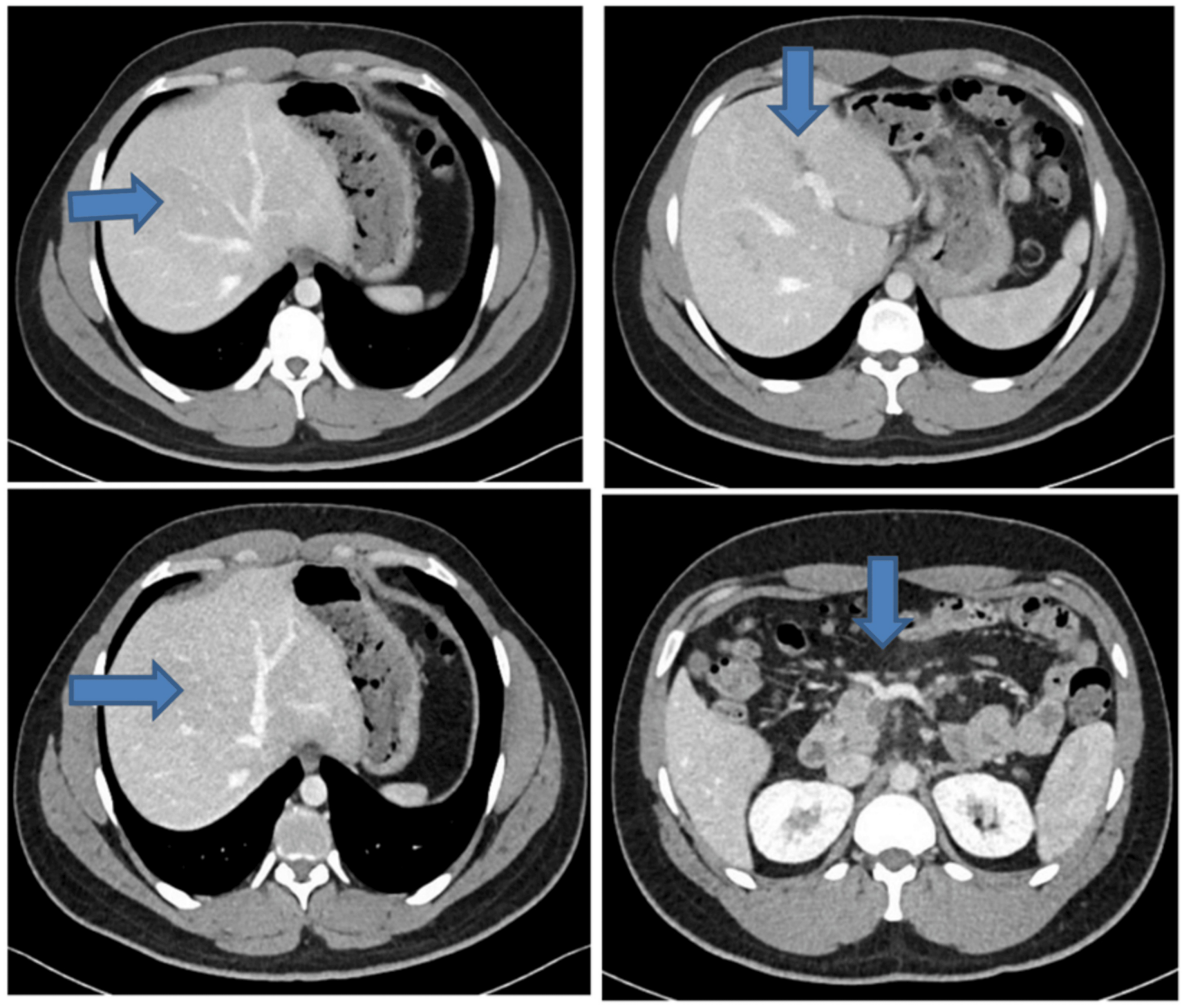
CT scan showing the filling defects of the right branch of the portal vein, extending to the splenic vein and the superior mesenteric vein, with complete non-opacification suggesting acute thrombosis.

A CT scan of the abdomen revealed filling defects within the right branch of the portal vein and its tributaries, extending to the confluence of the splenic vein and the superior mesenteric vein with complete non-opacification, with the appearance of acute thrombosis (Figure 1). There was no radiological or clinical evidence of cirrhosis or bowel ischemia. Further workup for hypercoagulable diseases or thrombophilia gave negative results, including JAK2 PCR (Table 1). It was hypothesized that clomiphene was a provoking factor for splanchnic vein thrombosis (SVT).

Subsequently, he was managed by therapeutic anticoagulation with low-molecular-weight heparin for 10 days and symptomatic treatment for pain relief during the hospital stay. His pain subsided before discharge, and his bowels returned to normal. Clomiphene was stopped by the infertility clinic after admission. He received a prescription for dabigatran 150 mg twice daily, which is a direct oral anticoagulant, for a period of six months upon discharge. The clinical decision regarding long-term anticoagulation was planned in association with the six-month follow-up MRI scans. A six-month follow-up MRI scan showed no major interval change with non-progressive but persistent thrombosis of the right portal vein and superior mesenteric vein. A decision was made to extend the duration of anticoagulation to 12 months, and the patient was referred for esophagogastroscopy to exclude the presence of esophageal varices.

## Discussion

To the best of our knowledge, this is the first reported case of PVT and SMVT linked to the use of clomiphene.

Testosterone therapy has been repeatedly associated with VTE [6], with a peak onset three months after commencing therapy [7]. This is the basis for hypothesizing that clomiphene therapy may also increase VTE risk. Clomiphene causes an increased release of the pituitary gonadotropins (luteinizing hormone and follicle-stimulating hormone), which, in turn, modulate the estrogen receptors in the hypothalamus, causing increased testosterone levels.

Clomiphene is generally regarded as a safe drug, with more common reported side effects of ovarian enlargement, vasomotor flushes, abdominal discomfort, nausea, and vomiting [1]; in men, the side effects include headache, dizziness, gynecomastia, testicular enlargement, and exacerbation of psychiatric illnesses [8]. VTE has only rarely been reported in association with clomiphene treatment, with one reported case of deep vein thrombosis (DVT) [9] and four cases of pulmonary embolism (PE) [2,3,5,10], each with a different duration of clomiphene treatment prior to the thrombosis, ranging from two days to two years. Isolated cortical vein thrombosis [4], cerebral sinus thrombosis [11], and central retinal vein occlusion [12-14] have also been reported in association with the use of clomiphene. The young age of our patient, lack of other identifiable provoking factors, and the contemporaneous use of clomiphene for three months make an association of VTE with clomiphene more likely.

The optimal duration or type of anticoagulation therapy for clomiphene-associated VTE is not known. If clomiphene is considered to be the provoking risk factor, then it is stopped, and standard duration anticoagulation for three months is reasonable for DVT or PE [15-17] and at least three to six months of anticoagulation for non-cirrhotic SVT, such as in this case [18,19]. For SVT, follow-up imaging is recommended to assess for the resolution of thrombus and guide duration [20]. Any testing for underlying hereditary or acquired thrombophilia with SVT should include JAK2 PCR due to the strong association [21].

## Conclusions

This case highlights a rare but significant adverse effect of CC therapy, with the development of PVT and SMVT in a young male patient. Although there are only a limited number of case reports on thrombosis associated with clomiphene, physicians should be aware of the potential for venous thrombosis. Caution should be used when prescribing clomiphene to patients with prior thrombosis or a strong family history of VTE. Clomiphene should be stopped if a patient presents with an acute thrombotic event. Clomiphene-associated thrombosis may occur at various sites, as shown in our case of PVT and SMVT.
